# Formation Mechanism for 2015/16 Super El Niño

**DOI:** 10.1038/s41598-017-02926-3

**Published:** 2017-06-07

**Authors:** Lin Chen, Tim Li, Bin Wang, Lu Wang

**Affiliations:** 1 Key Laboratory of Meteorological Disaster, Ministry of Education (KLME)/Joint International Research Laboratory of Climate and Environmental Change (ILCEC)/Collaborative Innovation Center on Forecast and Evaluation of Meteorological Disasters (CIC-FEMD), Nanjing University of Information Science and Technology, Nanjing, 210044 China; 2 International Pacific Research Center (IPRC), and Department of Atmospheric Sciences, SOEST, University of Hawaii at Manoa, Honolulu, HI 96822 USA

## Abstract

The extreme El Niño (EN) events in 1997/98 and 1982/83, referred to as super EN, exerted remarkable global influence. A super EN was anticipated on the way in early 2014 but failed to materialize toward the end of 2014. Whilst the scientific community was still puzzling about the cause of the aborted EN event in 2014, the remnants of the decaying warming in late 2014 unexpectedly reignited since February 2015 and grew into a super EN by the end of 2015. Understanding the onset mechanism of the 2015 EN event and its differences from past super EN events is crucial for improving EN prediction in a changing climate. Our observational analyses and modeling studies demonstrate that the principal difference between the 2015 EN and the past super ENs lies in exceptionally strong and consecutive occurrence of westerly wind burst events that turned around unfavorable ocean thermocline conditions in tropical western Pacific in early 2015, reigniting rapidly the surface warming in the eastern Pacific. By August the sea surface temperature anomalies reached a critical amplitude similar to that of the past super ENs; positive atmosphere-ocean feedbacks further amplify this warm episode into a super EN by the end of 2015.

## Introduction

The El Niño-Southern Oscillation (ENSO) is one of the greatest climate variabilities on the interannual timescale^1, 2^. Accurate prediction of ENSO, especially prediction of extreme El Niño (EN) such as those in 1982/83 and 1997/98 with extraordinary amplitudes of 4–5 °C warmer than normal (referred to as “super El Niño”^3, 4^), has great socioeconomic impacts^1, 2, 5, 6^. During 2014 and 2015, unusual development of warm episodes happened in the tropical Pacific^7^. In early 2014 many climate models predicted occurrence of a super EN by the end of 2014^8^, but such a forecast turned out to be a false alarm^7, 9^. When the scientific community were struggling with understanding the aborted super EN in 2014^10–14^, the unexpected emergence of the 2015 super EN event (hereafter 2015EN) brought the ENSO research community another surprise. Investigating the onset mechanism of the 2015 EN event and identifying the similarities and differences between 2015EN and the past super EN events are of great importance for improving our understanding of EN behaviors in a changing climate.

**Figure 1:**
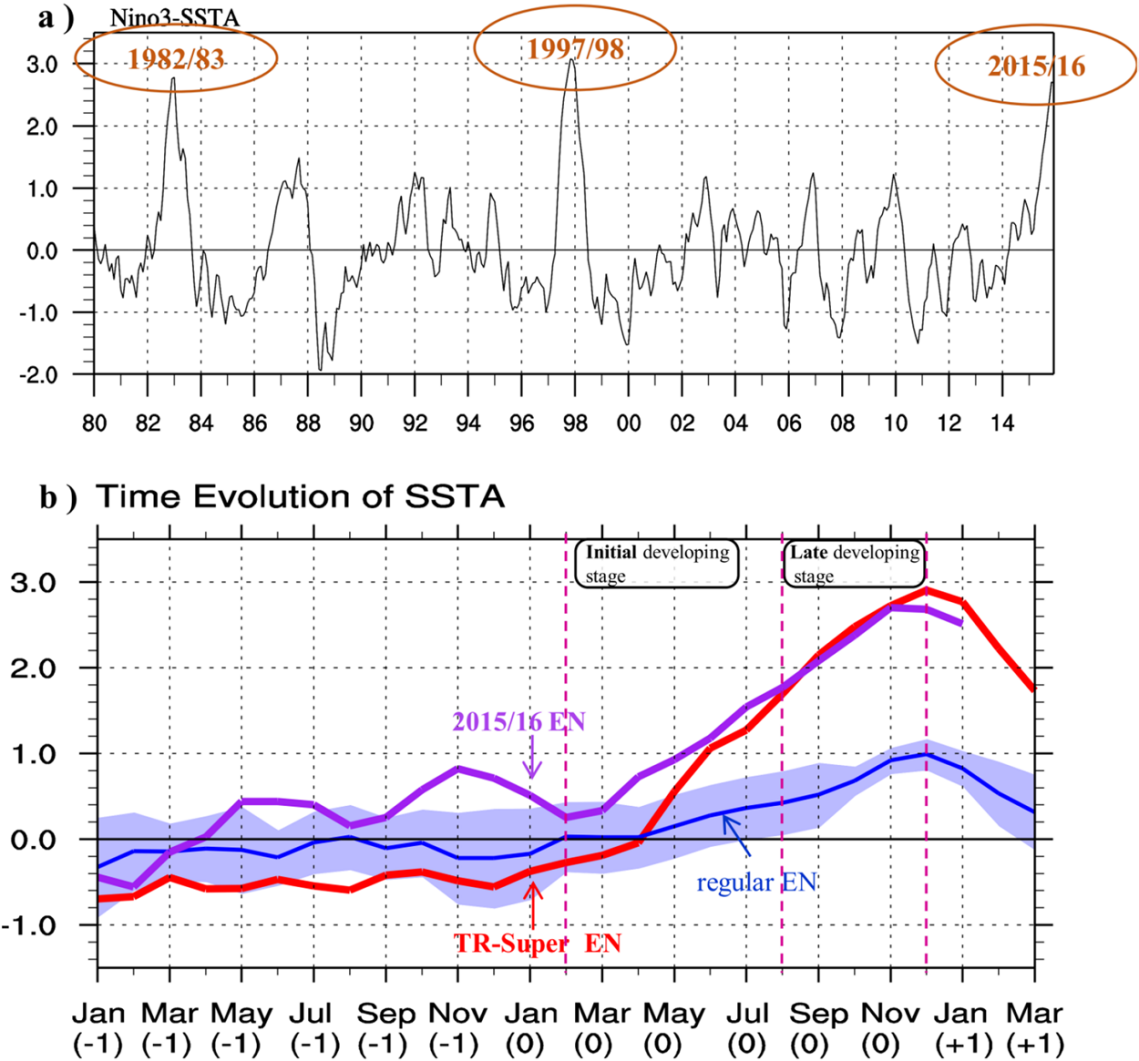
(**a**) Time series of the sea surface temperature anomaly (SSTA) averaged over Niño3 (i.e., Niño3 index) from ERSST. Here Niño3 region is bounded by 5°S-5°N and 150°W-90°W. (**b**) Temporal evolution of Niño3 SSTA. Purple line indicates the 2015/16 El Niño (i.e., 2015EN), red line indicates the composite of traditional super EN events (i.e., 1982/83 EN and 1997/98 EN), and the blue line indicates the composite of the regular EN events during 1980–2015 (including 1986/87, 1987/88, 1991/92, 1994/95, 2002/03, 2004/05, 2006/07 and 2009/10 ENs). The light blue shading indicates the inter-case spread, which is estimated with the inter-case standard deviation of the regular EN events. The magenta dashed lines divide the development of 2015EN into two stages, i.e., the initial developing stage (FMAMJJ[0]) and the late developing stage ([ASON[0]]). Here year[0] and year[−1] indicate the year of an EN event and the preceding year, respectively. This figure was generated by the NCAR Command Language (NCL, version 6.2.1, [Software]. (2014). Boulder, Colorado: UCAR/NCAR/CISL/VETS. http://dx.doi.org/10.5065/D6WD3XH5) and the licensed Microsoft PowerPoint.

As shown in Fig. 1a, the intensity of 2015EN during the mature phase (boreal winter) is comparable to the traditional super El Niño (hereafter TR-super EN, defined as the ensemble average of 1982 and 1997 super ENs due to their similarity in sea surface temperature anomaly (SSTA) evolution and structure). How was the 2015EN generated? Was the development of 2015EN similar to that in TR-super EN?

## Results

### Unfavorable ocean thermocline precondition of 2015EN

Figure 1b compares the evolutions of the Niño3 SSTA for 2015EN, TR-super EN and the composite of regular EN events during 1980–2015. Two marked differences are worth noting. Firstly, in contrast to TR-super EN that started from a weak cold episode in the preceding year, 2015EN was preceded by a weak warm episode in 2014 (Fig. 1b, Fig. S1). The weak warming peaked in November 2014 and then underwent gradual decay to the ensuing January 2015 (Fig. 1b). Secondly, in 2015EN a marked turnabout of the SSTA tendency (from negative to positive) happened around February 2015 (Fig. 1b). From February to July, both TR-super EN and 2015EN experience a rapid development, and we refer it as “initial developing stage”. By August 2015, the Niño3 SSTA reached 1.7 K, which is as strong as that of TR-super EN; in the late developing stage (i.e., August–November), the evolution of Niño3 SSTA in 2015EN appears to be similar to TR-super EN.

**Figure 2:**
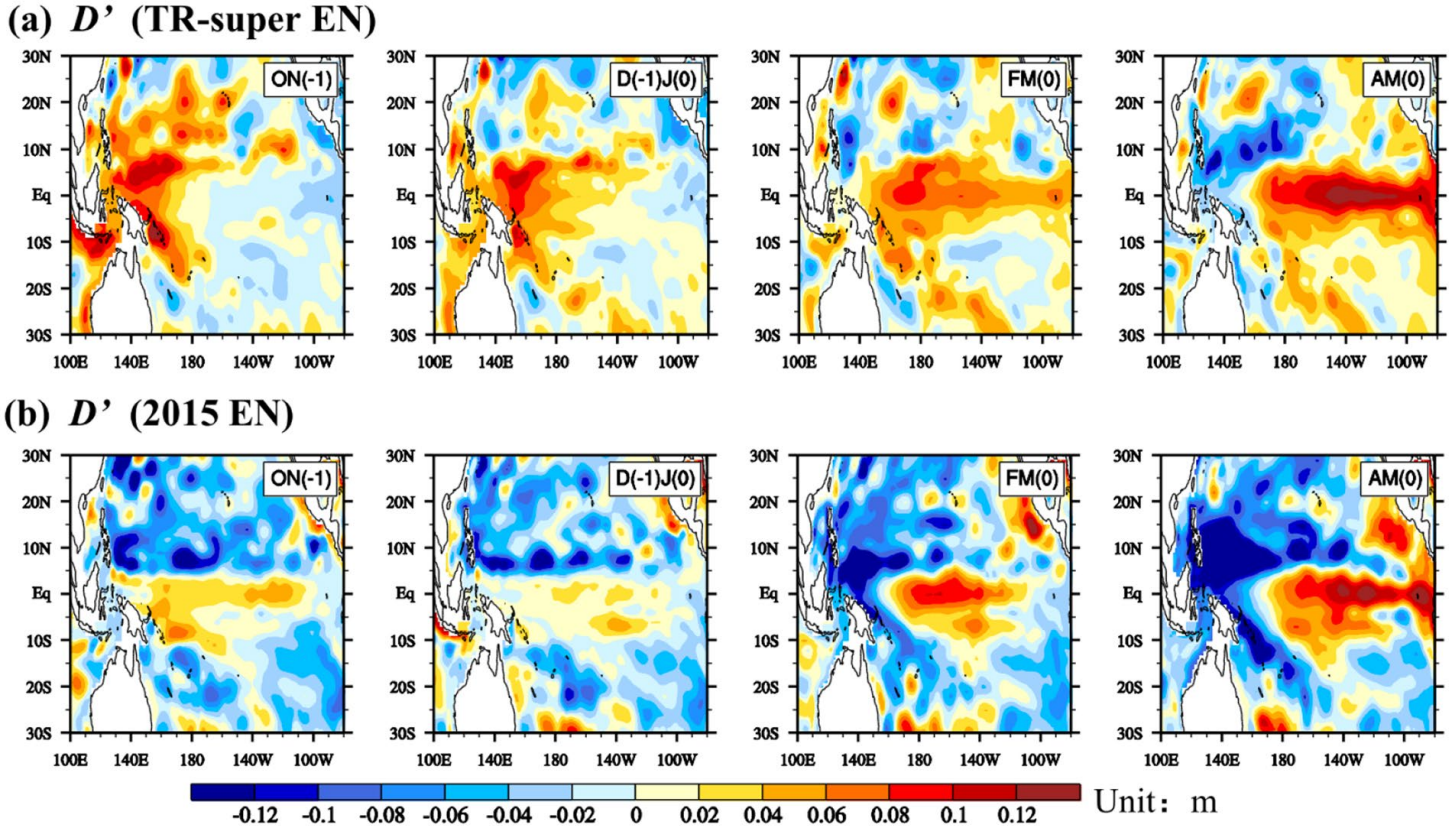
The evolution of the sea surface height anomaly (*SSH′*; a proxy of *D*′) from GODAS for ON[−1], D[−1]J[0], FM[0] and AM[0], derived from (**a**) the composite of TR-super EN and (**b**) 2015EN. Here a linear *D*′−*SSH′* relationship was applied. All plots were generated by the NCAR Command Language (NCL, version 6.2.1, [Software]. (2014). Boulder, Colorado: UCAR/NCAR/CISL/VETS. http://dx.doi.org/10.5065/D6WD3XH5).

During the initial developing stage, however, the precondition of thermocline depth anomaly (*D′*) and associated SSTA evolution differed markedly between 2015EN and TR-super EN. Note that distinctive precursory thermocline anomaly signals appeared in the off-equatorial western Pacific between 2015EN and TR-super EN (Fig. 2, Fig. S2). The ocean-atmosphere system prior to TR-super EN exhibited a La Niña state in OND[−1]J[0], as seen from Fig. 1b and Fig. S1b–d. Here year[0] and year[−1] indicate the year of an EN event and the preceding year, respectively. Equatorial easterly anomalies associated with the precursory cold anomaly caused anti-cyclonic wind stress curl anomalies, which built up positive upper-ocean heat content anomalies (i.e., positive *D′*) off the equator in ON[−1] (Fig. 2a). The positive off-equatorial *D*′ signals propagated westward as Rossby waves and became downwelling equatorial Kelvin waves after being reflected in the western boundary^15^. As seen in Fig. 2a, a positive *D*′ center appeared in central equatorial Pacific (CEP) in FM[0] and eastern equatorial Pacific (EEP) in AM[0]. It is the positive *D*′ at the equator that led to great thermocline and zonal advective feedbacks and thus a strong positive SSTA tendency during the initial developing stage of TR-super EN. By analyzing fourteen EN events among 1958–2008, Chen *et al*. (2016)^16^ reported that the positive *D*′ in tropical western Pacific during the pre-onset stage is significantly stronger in TR-super EN than regular EN events. Thus, a strong precursory positive *D*′ signal favored the initial rapid development of a super EN. A heat budget analysis (figure not shown) confirmed that the rapid warming during the initial developing stage of TR-super EN arose from the strong positive *D*′ at the equator, which can be traced back to the pre-onset condition of *D*′ over tropical western Pacific.

In contrast, the pre-onset condition of 2015EN was unfavorable for the occurrence of even a moderate EN event. During OND[−1]J[0], the ocean-atmosphere system possessed a weak and decaying EN pattern (Fig. 1b, Fig. S1a). A negative *D*′ built up over off-equatorial western Pacific during OND[−1]J[0] (Fig. 2b). The negative *D*′ was supposed to move to the equator in the following months, reducing the remnants of preceding positive thermocline anomalies at the equator. However, a positive *D*′ signal unexpectedly intensified over CEP in FM[0], and expanded into the EEP in AM[0]. The month-to-month evolution of 20 °C isotherm depth anomaly (a proxy of *D*′) from the Tropical Atmosphere Ocean (TAO) observation data further confirms this sudden emergence feature of *D*′ (Fig. S3).

The sudden emergence of this positive *D*′ center in CEP is responsible for the turnabout of the SSTA tendency in February 2015. This is supported by the mixed-layer heat budget shown in Fig. S4b. The major warming processes during MAM[0] were zonal advective feedback (term 1, $-u^{\prime} \partial \overline{T}/\partial x$) and thermocline feedback (term 5, $-\bar{w}\partial T^{\prime} /\partial z$), both of which were closely related to *D*′^17–19^. In contrast, the two terms were much weaker in D[−1]J[0], thus, the negative heat flux feedback term dominated the total SSTA tendency (Fig. S4a).

### Role of WWEs in the sudden emergence and continuous intensification of positive *D*′

**Figure 3:**
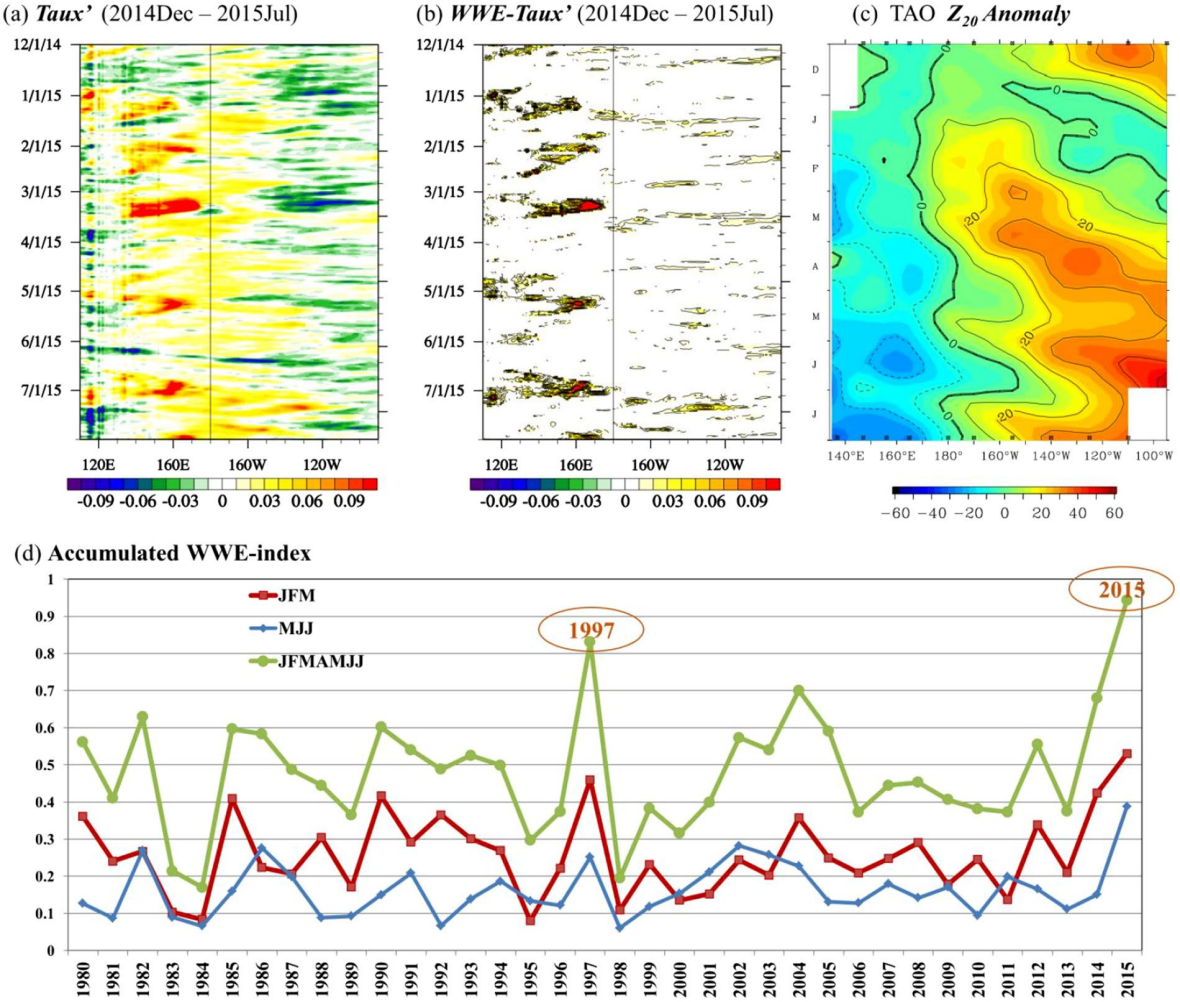
Evolution of (**a**) the zonal wind stress anomaly (*Taux*′), (**b**) *WWE-Taux*′, and (**c**) 20 °C isotherm depth anomaly along the equator, from Dec 1, 2014 to July 31, 2015. The *Taux*′ and *WWE-Taux*′ are derived from the zonal wind stress (*Taux*) daily data covering 1979–2015. See the main text for the detailed derivation method. Figure 3c is derived from the TAO/TRITON observation provided by PMEL. (**d**) Time series of the accumulated WWE-index, which is obtained through integrating the WWE-index for the period of January–March (JFM; red curve), May–July (MJJ; blue curve) and January–July (JFMAMJJ; green curve) of each year. The WWE-index in a given day is obtained by integrating the *WWE-Taux*′ over WWE region (see detailed description in Method). Figure 3a,b,c were generated by the NCAR Command Language (NCL, version 6.2.1, [Software]. (2014). Boulder, Colorado: UCAR/NCAR/CISL/VETS. http://dx.doi.org/10.5065/D6WD3XH5), and Fig. 3d was generated by the licensed Microsoft Excel.

The key to understand the sudden turnaround in February 2015 is to address what caused the sudden increase of *D*′ over CEP in early 2015. Previous studies suggested that high-frequency (HF) zonal wind forcing is important for the development of EN events^20–23^. The major component of HF zonal wind forcing was the so-called westerly wind events (hereafter WWEs^24–27^), which played a critical role in triggering EN or modulating EN amplitude^6, 10, 25–30^. Figure 3a shows the evolution of equatorial zonal wind stress anomaly (*Taux*′) in early 2015, which was obtained from raw daily zonal wind stress field (*Taux*) by subtracting the raw data from its climatologic seasonal cycle ($\bar{Taux}$). Note that there were a series of WWEs during the first three months of 2015 (i.e., JFM[0]) over western-central equatorial Pacific (WCEP; i.e., 5°S–5°N, 120°E–180°). The occurrence of the WWEs coincided with the sudden increase of *D*′ over CEP in FM[0] (Fig. 3c).

To quantitatively measure the strength of WWEs for each year, we introduce a new index that describes the accumulated effect of WWEs. Figure 3b shows the evolution of *WWE-Taux*′ along the equator. Here *WWE-Taux*′ represents the part of the HF component of *Taux*′ (named as *HF-Taux*′) that exceeds its climatologic standard deviation (see detailed description of these terms in Method). The pulses of *WWE-Taux*′ match well with the flaring up of *D*′ over CEP through January to July 2015 (Fig. 3b,c). During JFM 2015, there were three strong WWE episodes. The red curve in Fig. 3d shows the accumulated WWE index for January–March (JFM) of each year. It exhibits clearly that the overall intensity of the WWEs in JFM 2015 is the strongest from 1980 to 2015 (see red curve in Fig. 3d). In addition to the strong WWEs in JFM, the WWEs during May–July (MJJ) 2015 are also the strongest among the past 36 years (see blue curve in Fig. 3d). As a result, the overall accumulated intensity of WWEs in January–July 2015 is the most prominent among the 36 years (see green curve in Fig. 3d). Additionally, we calculated the WWE-index with a slightly modified definition, which confirms that the accumulated intensity of WWEs during January–July 2015 is the strongest among 1980–2015 (Fig. S5). Such an exceptionally strong WWE forcing during the first half of 2015 may play an essential role in the sudden emergence and continuous intensification of the positive *D*′ in CEP.

### Evidence from model experiments

To demonstrate the dynamic effect of *HF-Taux*′ in early 2015, oceanic general circulation model (OGCM) experiments were carried out. In the control (hereafter CNTL) run, the OGCM was integrated for 37 years, forced by the ERA-interim daily wind stress from 1979–2015. As shown in Fig. S6a, with realistic daily wind stress forcing, the OGCM is able to reproduce the interannual variability of SSTA in the Niño3 region similar to the observation. Parallel to the CNTL run, the sensitivity experiment is termed as “No-WWE” run, in which all the forcing fields were kept the same as the CNTL run except that the *HF-Taux*′ component over WWE regions was removed. The anomalous zonal wind stress forcing fields for both CNTL and No-WWE are shown in Fig. S6b,c.

**Figure 4:**
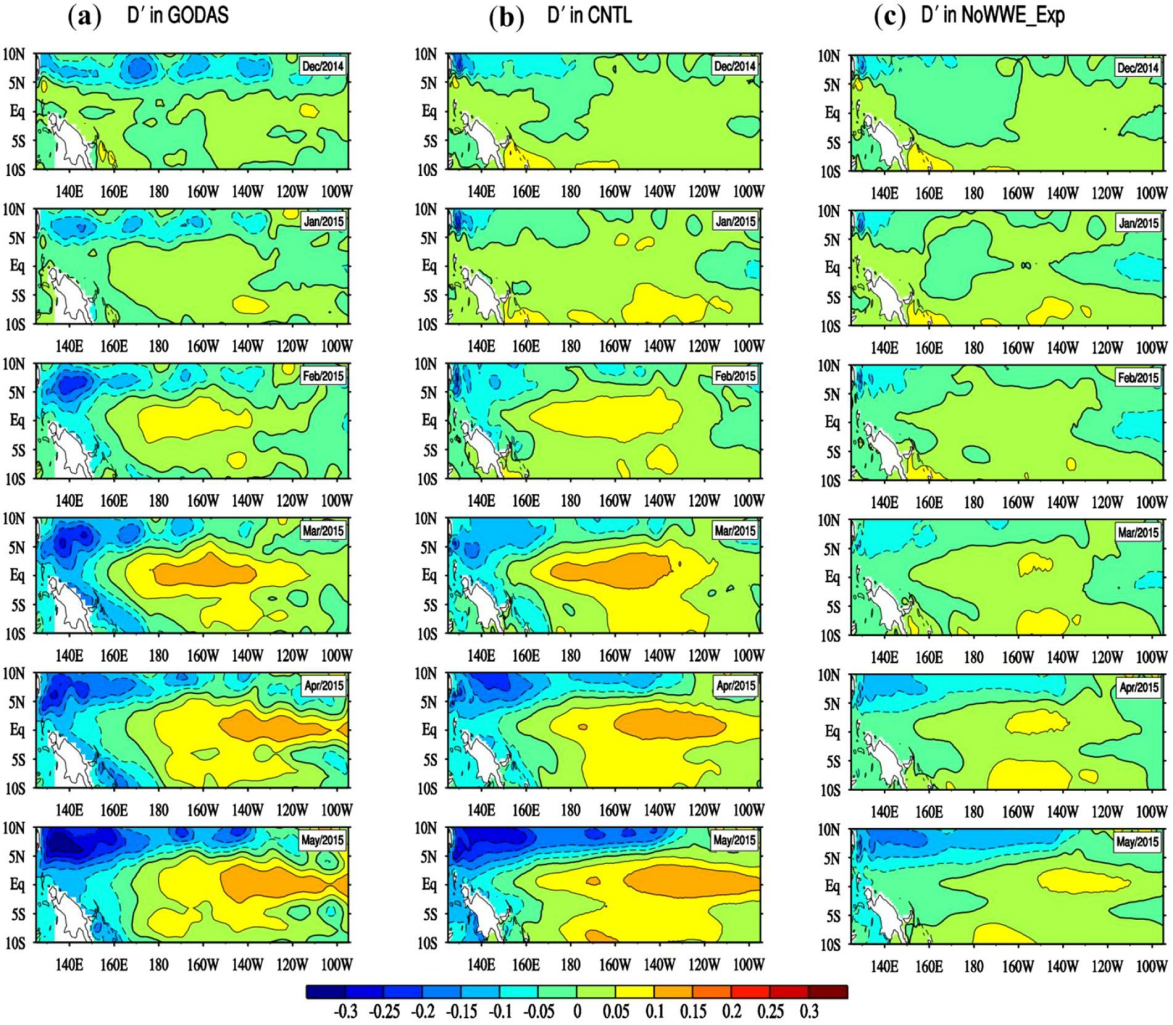
Month-to-month evolution of the sea surface height anomaly (unit: m; *SSH′*; a proxy of *D*′) from December 2014 to May 2015 in (**a**) GODAS, (**b**) CNTL run and (**c**) No-WWE run. All plots were generated by the NCAR Command Language (NCL, version 6.2.1, [Software]. (2014). Boulder, Colorado: UCAR/NCAR/CISL/VETS. http://dx.doi.org/10.5065/D6WD3XH5).

Figure 4 shows the evolution of sea surface height anomaly (*SSH′*, a proxy of *D*′) fields in the reanalysis (GODAS), the CNTL and No-WWE simulations. In the CNTL, the feature of *SSH′* evolution is generally similar to that depicted by GODAS, that is, a positive *SSH′* emerged over CEP in February 2015, and it intensified and propagated eastward in subsequent months. In contrast, the *SSH′* in the No-WWE run does not capture such a feature. The occurrence of a weak positive *SSH′* in May 2015 in the No-WWE experiment is a result of the interannual zonal wind stress anomaly forcing (which was a direct response to the warming in EEP). Thus, the OGCM experiments demonstrate that the WWEs in early 2015 exerted a dominant dynamic impact on the rapid emergence and intensification of the positive *D*′ center at the equator. The simulated equatorial SSTA in CNTL shows a similar evolution feature as the observed (Fig. S7a, Fig. S1a). In CNTL, the model is able to capture the warming in CEP in FM[0]. Subsequently, the warming continues to grow while gradually extending to the EEP in AM[0], which is consistent with the *D*′ evolution. In contrast, such a notable warming signal does not appear in the No-WWE run (Fig. S7b).

### Development of 2015EN in the late developing stage

Because of the accumulated dynamic effect of the exceptional WWEs, the SSTA in EEP increased rapidly and reached a critical value by August 2015, which was close to that of TR-super EN in August. Given such large SSTA amplitude, the SSTA may continue growing in northern fall through season-dependent positive air-sea feedback processes^31^. Next, we compared anomalous atmospheric and oceanic circulation patterns and the relevant air-sea feedback processes during the late developing stage (ASON[0]) between TR-super EN and 2015EN. A heat budget analysis result shows that the main contributors to the SSTA growth during this stage in 2015EN are zonal advective feedback term (term 1), thermocline feedback term (term 5), Ekman feedback term (term 4), and meridional advective feedback term (term 8), which are similar to those in TR-super EN (Fig. S8).

In fact, great similarities in 3-dimentional oceanic and atmospheric circulation anomalies occurred during ASON[0] between TR-super EN and 2015EN (see Figs S9 and 10). In the oceanic subsurface thermal structure, 2015EN exhibits a feature similar to that in TR-super EN, that is, the warm water piles up in EEP, anomalous mixed-layer currents flow toward the east, and warm (cold) subsurface temperature anomalies occur in central-eastern (western) equatorial Pacific. In the atmosphere, both TR-super EN and 2015EN show a consistent convection-circulation structure, that is, anomalous low-level westerlies and upper-level easterlies are approximately in phase with anomalous atmospheric convection in CEP. Thus it is concluded that the growth of 2015EN bore many similarities with that of TR-super EN during the late developing stage. Because of that, 2015EN finally evolved into a super EN by the end of 2015.

## Discussion

In summary, the occurrence of a series of exceptionally strong WWEs in early 2015 is the major driver to flare up a positive *D*′ center over CEP and cause the SSTA turnabout in February 2015. The accumulative forcing of the exceptionally strong WWEs caused the rapid growth of the *D*′ and thus SSTA at the equator, and by August the amplitude of the SSTA had reached a critical value similar to that in TR-super EN. Afterwards, positive air-sea feedbacks continued strengthening the SSTA, leading to the formation of a super EN by the end of 2015.

**Figure 5:**
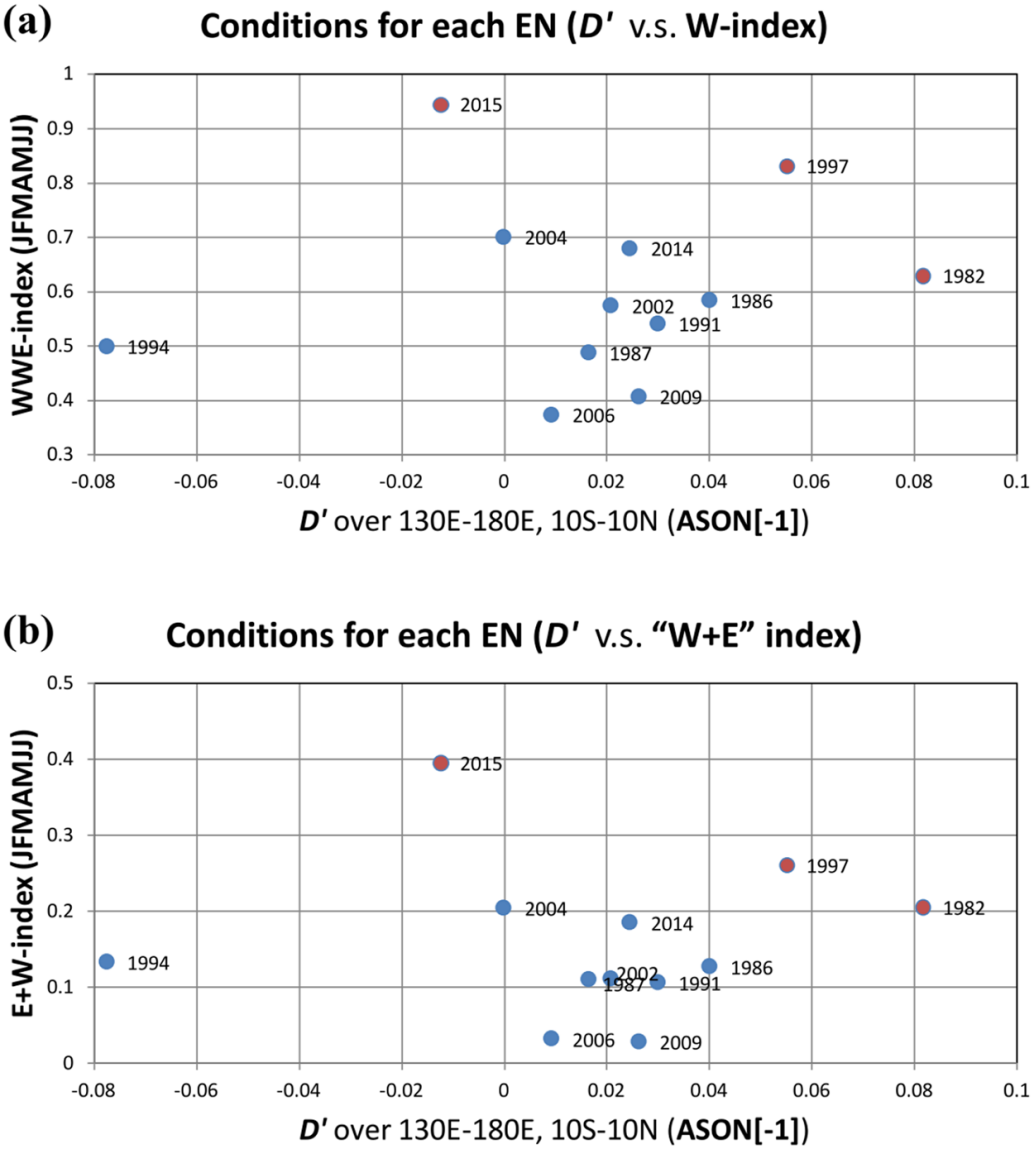
(a) Scatterplot of the Jan–Jul (0) accumulated WWE index and the precursory *D*′ signal for each El Niño event during 1979–2015. Here the precursory *D*′ signal is estimated by the Aug–Nov(−1) averaged sea surface height anomaly (a proxy of *D*′) over the tropical western Pacific (130°E–180°, 10°S–10°N). **(b**) same as (**a**) but for the accumulated “W + E” index, which is the summation of the accumulated WWE and accumulated EWE index. It shows that when taking into account the role of easterlies, the spreading between 2015 EN and other ENs revealed by the “W + E” index (Fig. 5b) resembles that revealed by the WWE index alone (Fig. 5a). The specific calculation of the accumulated WWE, EWE, and “W + E” index is introduced in the method section. This figure was generated by the licensed Microsoft Excel.

The unique developing characteristic in 2015 breaks our traditional view of El Niño formation, which emphasized the importance of the pre-conditional thermocline condition. For instance, as shown in Fig. 5, almost all EN events since 1979 were preceded by a positive precursory *D*′ signal (except the 1994 event, which was triggered by extratropical SSTA, see the work by Su *et al*. (2014)^17^ for details), whereas the 2015 event came as an exception: a negative, rather than a positive, precursory *D*′ appeared over the western Pacific. Here the magnitude of the precursory *D*′ signal is estimated by the Aug–Nov[−1] averaged *SSH′* over tropical western Pacific (130°E–180°, 10°S–10°N). It has been shown (e.g., Chen, M *et al*. 2016^32^) that almost all El Niños since 1979 (except 1986 event) had a quick phase transition to either La Niña or a normal state after the peak winter, regardless of the strength of the El Niños. Given that 2014 is a weak El Niño year, one would expect that either a La Niña or a normal state would emerge in the subsequent winter. Unexpectedly, a super El Niño occurred by the end of 2015. The ocean mixed layer budget analyses and idealized numerical modeling experiments in this study demonstrated that the consecutive extremely strong WWEs in 2015 indeed were able to turn around the unfavorable ocean thermocline conditions and lead to the development of a super EN. The marked contrast of precursory *D*′ signal between the 2015 EN and the traditional super ENs (Fig. 5) suggests two distinctive routes for super El Niño formation. In the first route, a key element is the occurrence of exceptionally strong positive precursory *D*′; and this strong precursory *D*′ signal was accompanied by either moderate WWEs (e.g., in 1982) or relatively strong WWEs (e.g., in 1997) (Fig. 5a). In the second route, a key element is the occurrence of exceptionally strong WWEs, even though the precursory *D*′ signal is quite weak or even negative. An example of this second scenario is the 2015 event. Thus, to accurately predict ENSO, we need to monitor HF WWE activities and assimilate these HF WWE activities into operational forecast models. An interesting question is what caused the WWE events in early 2015. A recent study^33^ suggested that the first burst of WWE in early spring of 2015 was triggered by the Arctic Oscillation (AO) event^34^, and was further strengthened by the Madden-Julian Oscillation activity and northerly cold surges from East Asia–western Pacific. Chen, SF *et al*. (2016)^33^ also suggested that the subsequent WWE in May 2015 was possibly induced by southerly surges from the Australian monsoon. Further observational and modeling studies are needed to confirm the results above.

An alternative explanation for the absence of strong and positive *D*′ signal prior to 2015 EN is that the precursory *D*′ signal is not particularly predictive for extreme EN events. This result seems consistent with the result of McPhaden (2003)^35^ and McPhaden (2012)^36^, who suggested that the lead time between the upper ocean heat content signal and ENSO peak phase decreased from 2–3 seasons in the period of 1980–1999 to only one season in the first decade of 21^st^ century. It is worth mentioning that a recent study by Levine and McPhaden (2016)^9^ (hereafter LM2016) suggested that the episodic easterly wind events in July 2014 (Hu and Fedorov 2016) not only inhibited the development of 2014 EN but also recharged warm water volume (WWV^37^), based on the argument that the heat content anomaly can remain in place for an extended duration (McGregor *et al*. 2016^38^). ﻿LM2016 argu﻿ed the resultant enhanced WWV anomaly gave 2015/16 EN a head start, which, combined with the strong WWEs in 2015, ultimately resulted in a super EN event of 2015/16. The role of the WWEs in helping pushing the EN’s growth in the present study is to a large extent in agreement with LM2016. The difference lies in that the present study considers the precursory *D*′ over tropical western Pacific as the precondition for an EN event (largely from the view of the delayed oscillator^15, 39^), and pointed out that the negative *D*′ is unfavorable for 2015 EN’s growth, whereas LM2016 considered the WWV or zonal mean *D*′ as the precondition (largely from the view of the recharge/discharge paradigm^40^), and pointed out that the WWV is favorable for 2015 EN’s growth. Note that zonal mean *D*′ at the equator dropped quickly in late 2014 and reached nearly negative value by January 2015 (purple cure in Fig. S11), while zonally integrated warm water volume anomaly at the equator shows a persistent positive anomaly throughout 2014 and 2015 (as reported by the TAO/PMEL online data, see red curve in Fig. S11). It is also noted that the WWV anomaly derived from GODAS (green curve in Fig. S11) shows a sharp decrease from September 2014 to January 2015 and reaches a small magnitude in January 2015, which differs from the TAO-derived WWV anomaly. The discrepancy of the precursory signals between zonal mean *D*′ and WWV calls for further studies. It is not clear why they are quite consistent in most of past EN events but different in 2014–15.

## Data and Methods

### Data

The observational sea surface temperature dataset used in this study is from the National Oceanic and Atmospheric Administration Extended Reconstructed Sea Surface Temperature version 3b (ERSSTv3b^41^). A 20 °C isotherm depth dataset is obtained directly from the Pacific Marine Environmental Laboratory (PMEL) Tropical Atmosphere Ocean (TAO)/Triangle Trans Ocean Buoy Network (TRITON) data and visualization service (see website at http://www.pmel.noaa.gov/tao/jsdisplay/). The zonal winds at 850hPa and 200hPa are derived from the ECMWF reanalysis product (ERA-interim^42^). The surface heat flux data are from the NCEP reanalysis version 2 (NCEPv2^43^). The daily surface wind stress from both ERA-interim and NCEP2 are used in this study. The NCEP Global Ocean Data Assimilation System (GODAS^44^) provides the oceanic 3-dimensional temperature and velocity fields, and sea surface height fields. The precipitation data is from the Climate Prediction Center Merged Analysis of Precipitation (CMAP^45^).

### Mixed layer heat budget analysis

A mixed layer heat budget analysis is performed to diagnose the specific dynamic and thermodynamic processes in contributing to the SSTA growth during the ENSO developing phase. The mixed-layer temperature tendency equation^16–19, 46^ is

1 $$\begin{array}{ccc}{\rm{\partial }}{T}^{^{\prime} }/{\rm{\partial }}t & = & -{u}^{^{\prime} }{\rm{\partial }}\bar{T}/{\rm{\partial }}x-\bar{u}{\rm{\partial }}{T}^{^{\prime} }/{\rm{\partial }}x-{u}^{^{\prime} }{\rm{\partial }}{T}^{^{\prime} }/{\rm{\partial }}x-{w}^{^{\prime} }{\rm{\partial }}\bar{T}/{\rm{\partial }}z-\bar{w}{\rm{\partial }}{T}^{^{\prime} }/{\rm{\partial }}z-{w}^{^{\prime} }{\rm{\partial }}{T}^{^{\prime} }/{\rm{\partial }}z\\  &  & \quad {\rm{t}}{\rm{e}}{\rm{r}}{\rm{m}}\,1\qquad \,\,{\rm{t}}{\rm{e}}{\rm{r}}{\rm{m}}\,2\qquad \,\,\,{\rm{t}}{\rm{e}}{\rm{r}}{\rm{m}}\,3\,\,\,{\rm{t}}{\rm{e}}{\rm{r}}{\rm{m}}\,4\qquad \,\,\,{\rm{t}}{\rm{e}}{\rm{r}}{\rm{m}}\,5\,\,\,{\rm{t}}{\rm{e}}{\rm{r}}{\rm{m}}\,6\\  &  & -{v}^{^{\prime} }{\rm{\partial }}\bar{T}/{\rm{\partial }}y-\bar{v}{\rm{\partial }}{T}^{^{\prime} }/{\rm{\partial }}y-{v}^{^{\prime} }{\rm{\partial }}{T}^{^{\prime} }/{\rm{\partial }}y+\frac{{{Q}^{^{\prime} }}_{{\rm{n}}{\rm{e}}{\rm{t}}}}{\rho {C}_{p}H}+R\\  &  & \quad {\rm{t}}{\rm{e}}{\rm{r}}{\rm{m}}\,7\qquad \,\,{\rm{t}}{\rm{e}}{\rm{r}}{\rm{m}}\,8\qquad \,\,{\rm{t}}{\rm{e}}{\rm{r}}{\rm{m}}\,9\quad \quad \,{\rm{t}}{\rm{e}}{\rm{r}}{\rm{m}}\,10\end{array}$$

where *u, v*, and *w* are the three-dimensional oceanic current; *T* represents the mixed-layer temperature; ()’ denotes the interannual anomaly variables; (−) denotes the climatologic mean variables; *Q*_*net*_ denotes the sum of net downward shortwave radiation absorbed in mixed-layer (*Q*_*sw*_), surface net downward longwave radiation, and surface sensible and latent heat fluxes (the positive sign represents that the ocean receives heat); *R* indicates the residual term; *C*_*p*_ and *ρ*_*o*_ are the specific heat of seawater and the density of seawater, respectively; and *H* is the mixed-layer depth that varies in time and space. *H* is defined as the depth where ocean temperature is 0.8 °C lower than the surface, following Saha *et al*. (2006)^44^ and Wang *et al*. (2012)^46^. All the budget terms in equation (1) are integrated from the surface to the mixed-layer depth. Considering the penetration of shortwave beyond mixed-layer, *Q*_*sw*_ is estimated as (Wang *et al*., 2012)^46^

2 $${Q}_{{\rm{sw}}}={Q}_{{\rm{surf}}}-0.47{Q}_{{\rm{surf}}}{e}^{-0.04H}$$

where ${Q}_{surf}$ is net downward surface shortwave radiation.

### Accumulated WWE index, EWE index and “W + E” index

First, a Lanczos bandpass filter^47^ is applied to extract the HF component (less than 90 days) of the *Taux*′ (hereafter *HF-Taux*′) from the 37-year (1979–2015) daily data. Then a climatological standard deviation field [i.e., *HF-Std(i, j, d)*] is calculated based on the *HF-Taux*′(*i, j, d, y*) among the 37 years, where “(*i, j*)” indicates a grid point, and “*d*” and “*y*” respectively indicate day and year. For a given day, the region where the *HF-Taux*′ is greater than *one HF-Std* is defined as the WWE region, and the exceeding part is referred to as *WWE-Taux*′ (i.e., $WWE \mbox{-} Tau{x}^{^{\prime} }=HF \mbox{-} Tau{x}^{^{\prime} }\,minus\,HF \mbox{-} Std$); the region where the *HF-Taux*′ is less than *negative one HF-Std* is defined as the EWE region, and the exceeding part is referred to as *EWE-Taux*′ (i.e., $EWE \mbox{-} Tau{x}^{^{\prime} }=HF \mbox{-} Tau{x}^{^{\prime} }\,minus\,(negative\,HF \mbox{-} Std)$). In the western-central equatorial Pacific (WCEP; i.e., 5°S-5°N, 120°E-180°), for a given day one may obtain the WWE index through integrating *WWE-Taux*′ over the WWE region (i.e., $WWE \mbox{-} index(d)=\frac{\oint WWE \mbox{-} Tau{x}^{^{\prime} }(i,j,d)dS}{{S}_{total}}$) and obtain the EWE index through integrating *EWE-Taux*′ over the EWE region (i.e., $EWE \mbox{-} index(d)=\frac{\oint EWE \mbox{-} Tau{x}^{^{\prime} }(i,j,d)dS}{{S}_{total}}$). Here “*d*” indicates a given day, “*dS*” indicates the grid cell area of a grid point (*i*, *j*), and *S*_*total*_ indicates the total area of WCEP. The accumulated WWE-index and the accumulated EWE-index can be obtained further through respectively integrating the WWE-index and EWE-index for a certain period. For example, through calculating the summation of the WWE-index from January to July for each year, one may obtain the Jan–Jul accumulated WWE index year by year. The accumulated “W + E” index is the summation of the accumulated WWE and accumulated EWE index.

An alternative WWE index (hereafter WWE-index2) is calculated based on the same procedure except that the *HF-Std(i, j)* is calculated based on daily data during the entire analysis period so that a constant value of *HF-Std* is used for each day.

Two independent daily *Taux* datasets (i.e., ERA-interim and NCEP2) are employed to obtain the accumulated WWE index and EWE index, respectively; and the ensemble average of them are used in this study.

### Ocean global circulation model (OGCM)

The OGCM used in this study is LICOM2.0^48^, which is the oceanic component of the climate system models of both FGOALS-s2^49^ and FGOALS-g2^50^ that participated CMIP5. Its horizontal resolution is 0.5° in tropical region. Vertically it has 30 levels with 10 m resolution in upper 150 m. For details, please refer to Liu *et al*. (2012)^48^.

### Graphic software

All maps and plots were produced using the NCAR Command Language (NCL, version 6.2.1, [Software]. (2014). Boulder, Colorado: UCAR/NCAR/CISL/VETS. http://dx.doi.org/10.5065/D6WD3XH5), except that Figs 1, S9 were additionally modified by licensed Microsoft PowerPoint and Figs 3d, 5, S5, S11 were generated by the licensed Microsoft Excel.

## Electronic supplementary material

Supplementary Material
