# Supplementary material for: Formation Mechanism for 2015/16 Super El Niño
Source: Sci Rep. 2017 Jun 7;7:2975. doi: 10.1038/s41598-017-02926-3 (PMC5462824; doi:10.1038/s41598-017-02926-3)
Supplement: Supplementary file 1 — Supplementary Material [file 41598_2017_2926_MOESM1_ESM.pdf]

Supplementary Material for

Formation Mechanism for 2015/16 Super El Niño

Lin Chen[1,2], Tim Li[1,2], Bin Wang[1,2], Lu Wang[1,2]

1 Key Laboratory of Meteorological Disaster, Ministry of Education (KLME)/Joint International Research Laboratory of Climate and Environmental Change (ILCEC)/Collaborative Innovation Center on Forecast and Evaluation of Meteorological Disasters (CIC-FEMD), Nanjing University of Information Science and Technology, Nanjing, 210044, China

2 International Pacific Research Center (IPRC), and Department of Atmospheric Sciences, SOEST, University of Hawaii at Manoa, Honolulu, HI 96822, USA

Submitted to *Scientific Reports*

This PDF file includes:

Supplementary Figs. S1 to S11

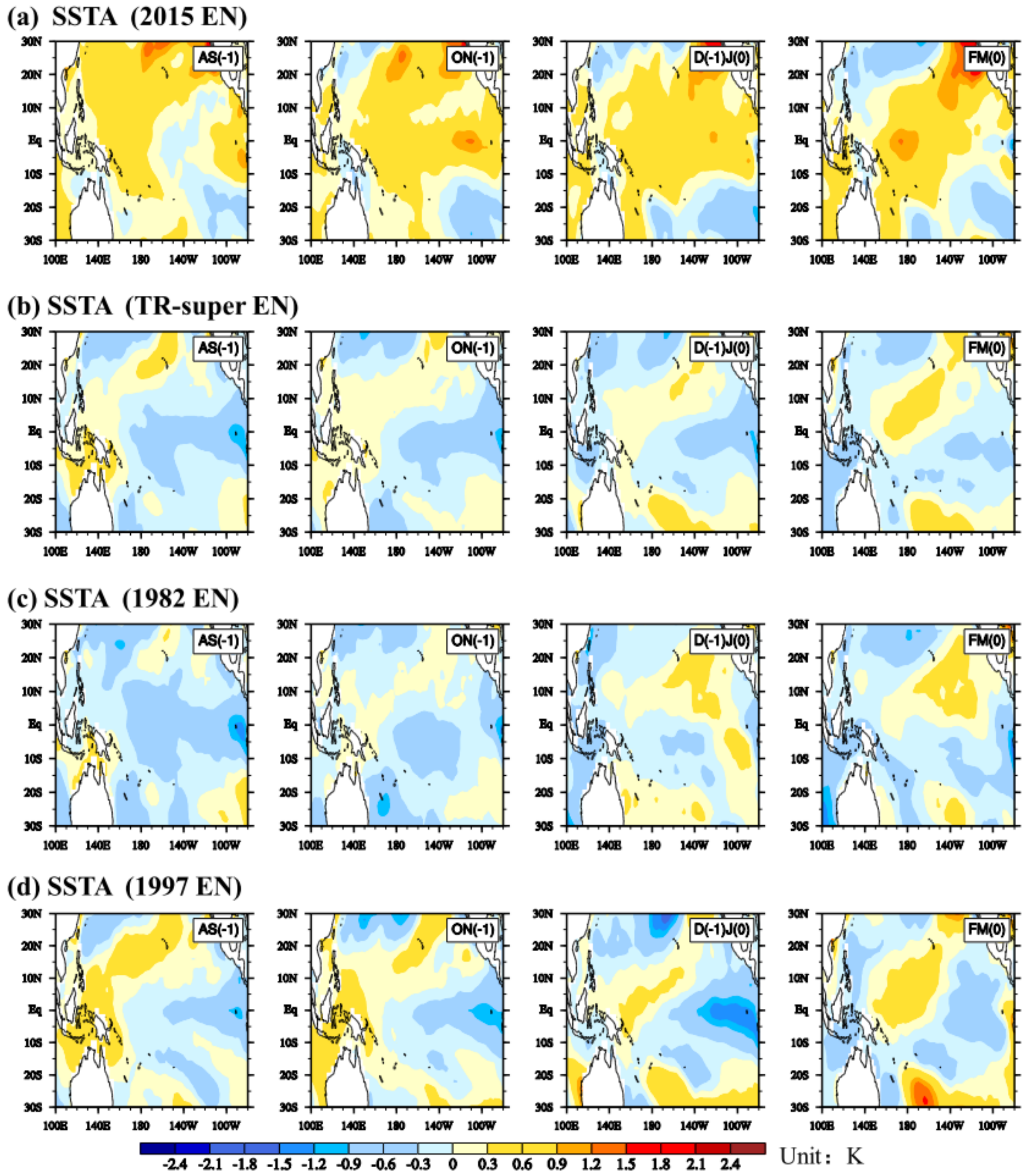

Figure S1. The evolution of the observed sea surface temperature anomaly (unit: K) for AS[−1], ON[−1], D[−1]J[0], and FM[0], which are derived from (**a**) 2015 EN, (**b**) the composite of TR-super EN, (**c**) 1982EN and (**d**) 1997EN. All plots were generated by the NCAR Command Language (NCL, version 6.2.1, [Software]. (2014). Boulder, Colorado: UCAR/NCAR/CISL/VETS. http://dx.doi.org/10.5065/D6WD3XH5).

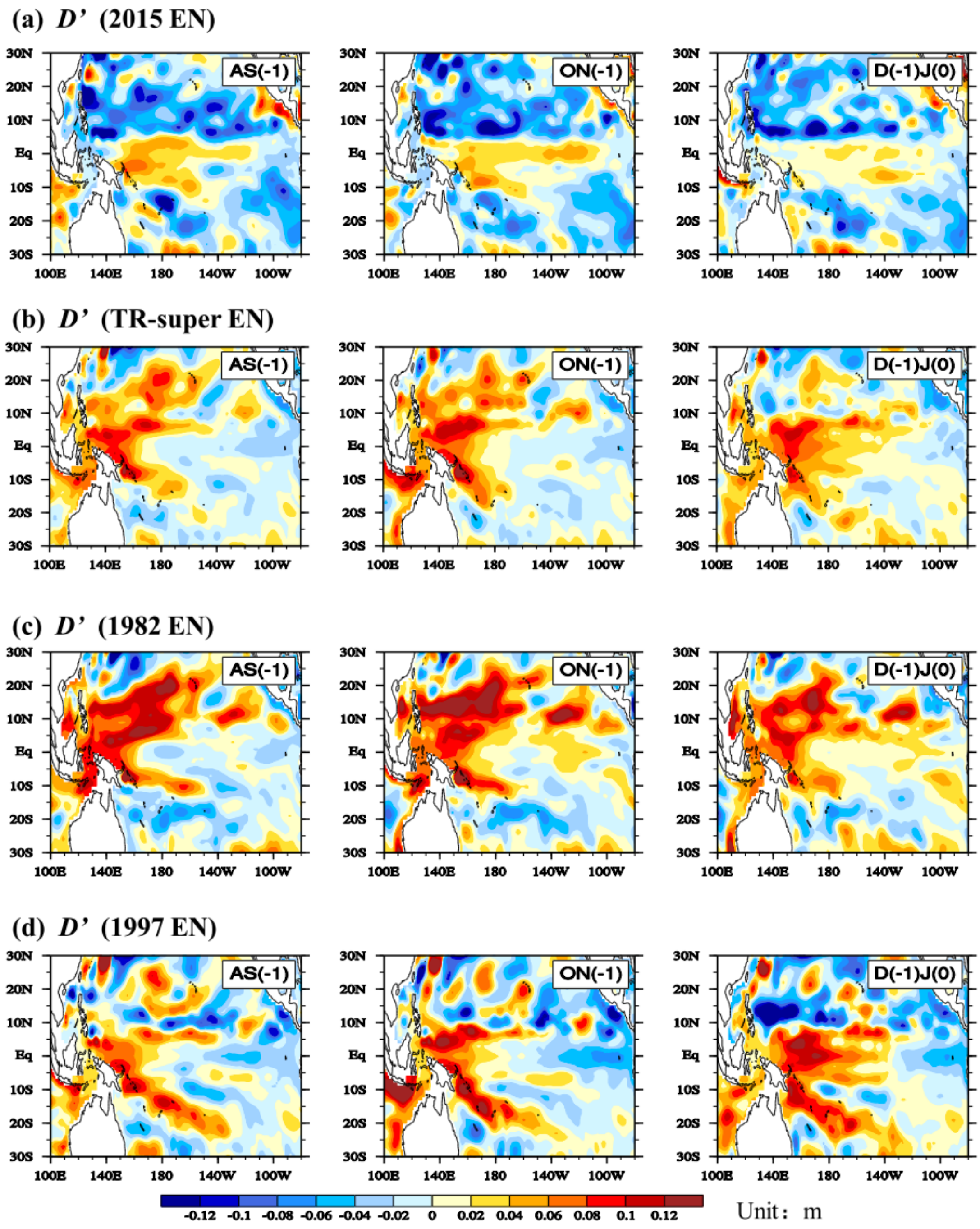

Figure S2. The evolution of the observed sea surface height anomaly (*SSH’*; a proxy of *D’*) for AS[−1], ON[−1], and D[−1]J[0], which are derived from (**a**) 2015 EN, (**b**) the composite of TR-super EN, (**c**) 1982EN and (**d**) 1997EN. Here a linear *D’*–*SSH’* relationship was applied. All plots were generated by the NCAR Command Language (NCL, version 6.2.1, [Software]. (2014). Boulder, Colorado: UCAR/NCAR/CISL/VETS. http://dx.doi.org/10.5065/D6WD3XH5).

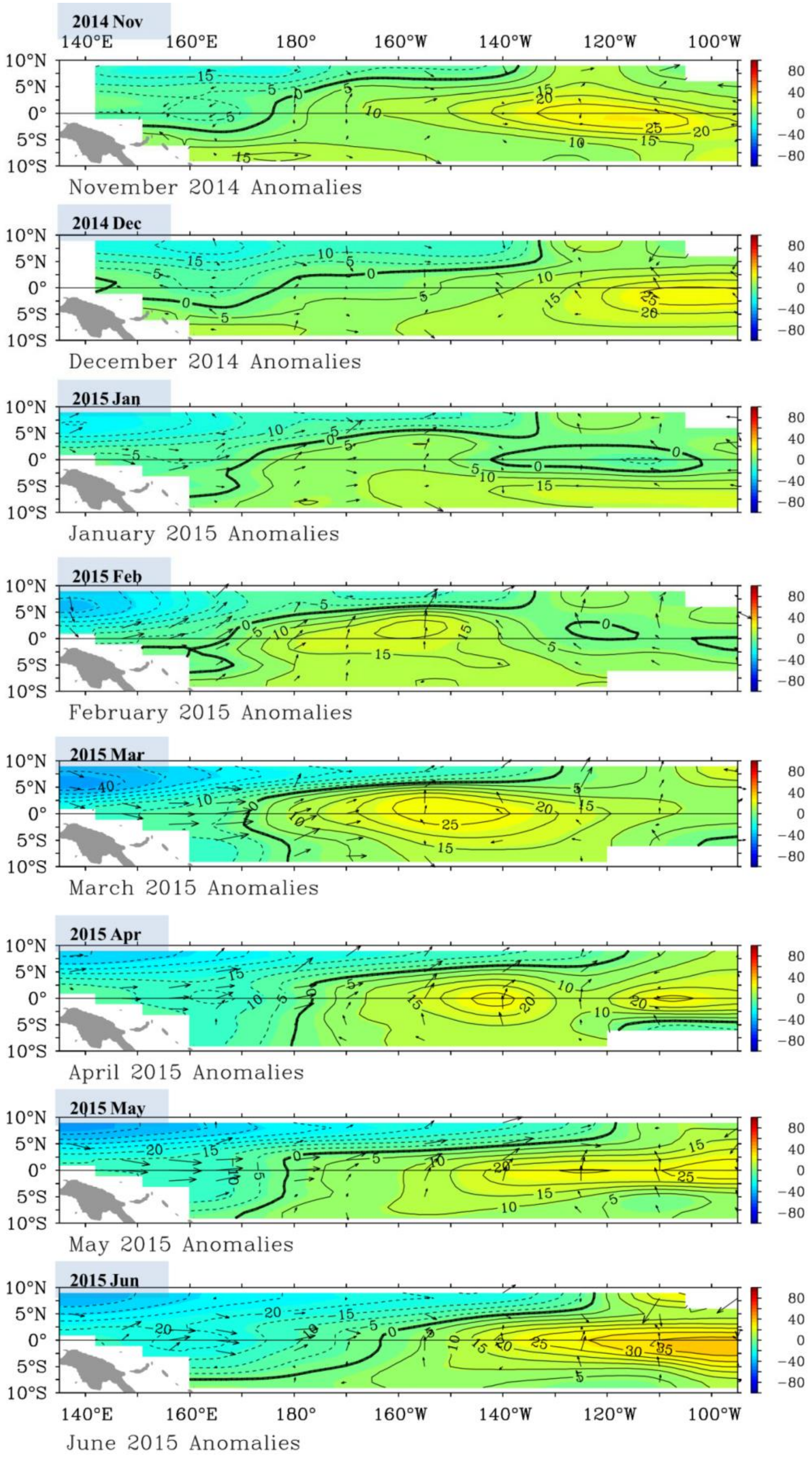

Figure S3. Month-to-month evolution of the 20 ℃ isotherm depth anomaly (also a proxy of *D'*) from November 2014 to June 2015. The 20 ℃ isotherm depth anomalies are directly derived from the TAO/TRITON observation provided by PMEL (http://www.pmel.noaa.gov/tao/jsdisplay). All plots were generated by the NCAR Command Language (NCL, version 6.2.1, [Software]. (2014). Boulder, Colorado: UCAR/NCAR/CISL/VETS. http://dx.doi.org/10.5065/D6WD3XH5).

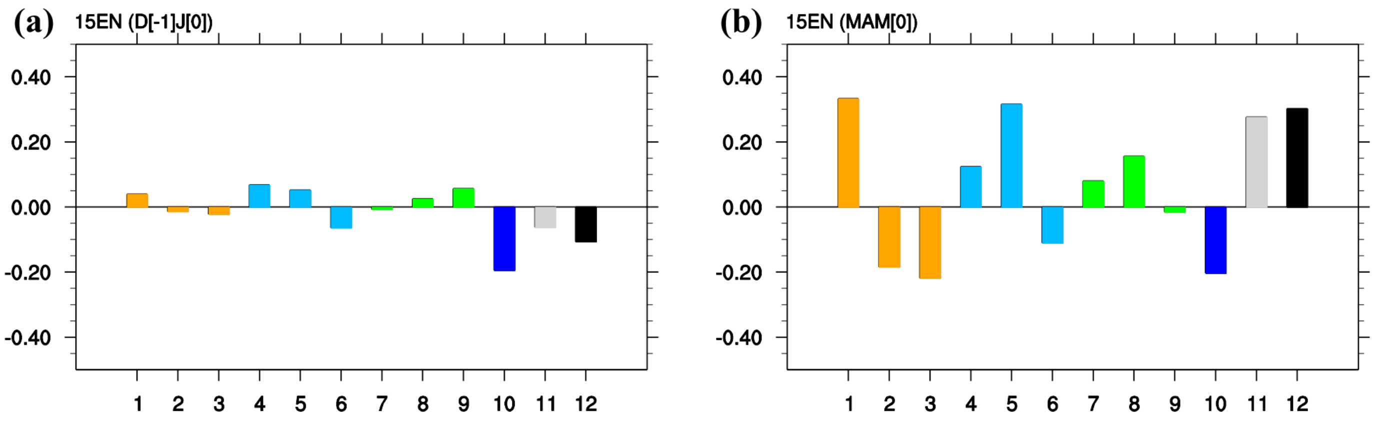

Figure S4. The mixed-layer heat budget terms (unit: K/month) for Niño3 SSTA during (**a**) the period from December 2014 to January 2015 (D[-1]J[0]) and (**b**) the period from March to May 2015 (MAM[0]). Bar 12 denotes the mixed-layer temperature tendency $\partial T'/\partial t$, and bar 11 is the sum of all first 10 terms. The remaining terms are indicated by bar 1: $-u'\partial\overline{T}/\partial x$, bar 2: $-\overline{u}\,\partial T'/\partial x$, bar 3: $-u'\partial T'/\partial x$, bar 4: $-w'\partial\overline{T}/\partial z$, bar 5: $-\overline{w}\,\partial T'/\partial z$, bar 6: $-w'\partial T'/\partial z$, bar 7: $-v'\partial\overline{T}/\partial y$, bar 8: $-\overline{v}\,\partial T'/\partial y$, bar 9: $-v'\partial T'/\partial y$, and bar 10: $Q'_{net}/\rho_o C_p H$. See the mixed-layer temperature tendency equation in the method section for more details. All plots were generated by the NCAR Command Language (NCL, version 6.2.1, [Software]. (2014). Boulder, Colorado: UCAR/NCAR/CISL/VETS. http://dx.doi.org/10.5065/D6WD3XH5).

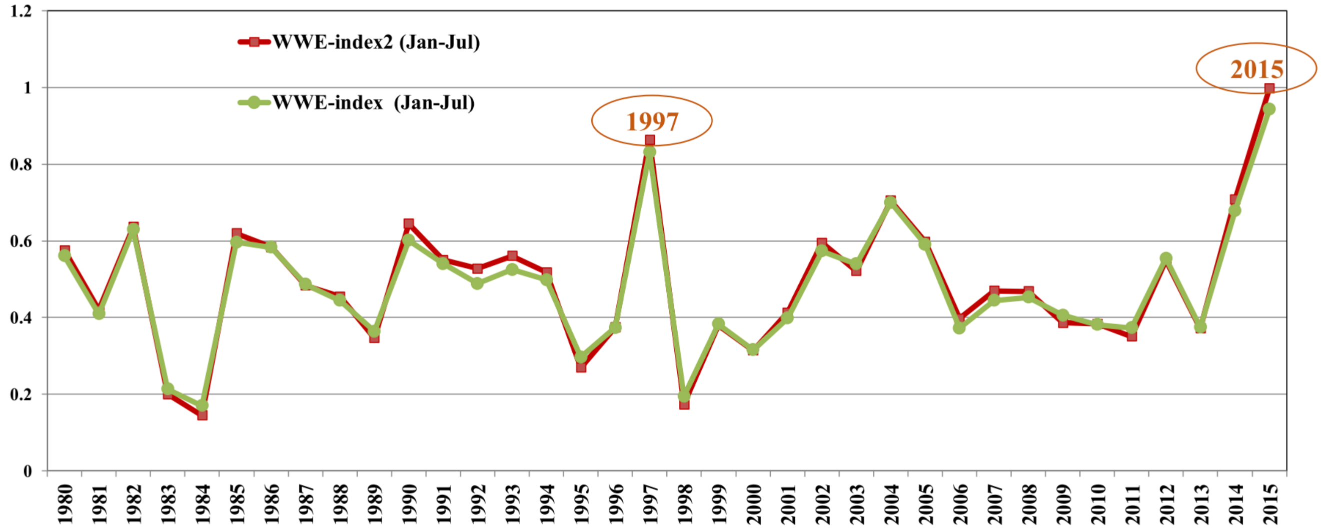

Figure S5. Comparision of the WWE-index with different definitions. The green line shows the time series of the accumulated WWE-index for January-July (JFMAMJJ). For convenient comparison, such green line is same as that shown in Figure 3d and is obtained as introduced in Method. The red line is same as the green line except for the accumulated WWE-index2, which is obtained by using a slightly modified definition (refer to Method). This figure was generated by the licensed Microsoft Excel.

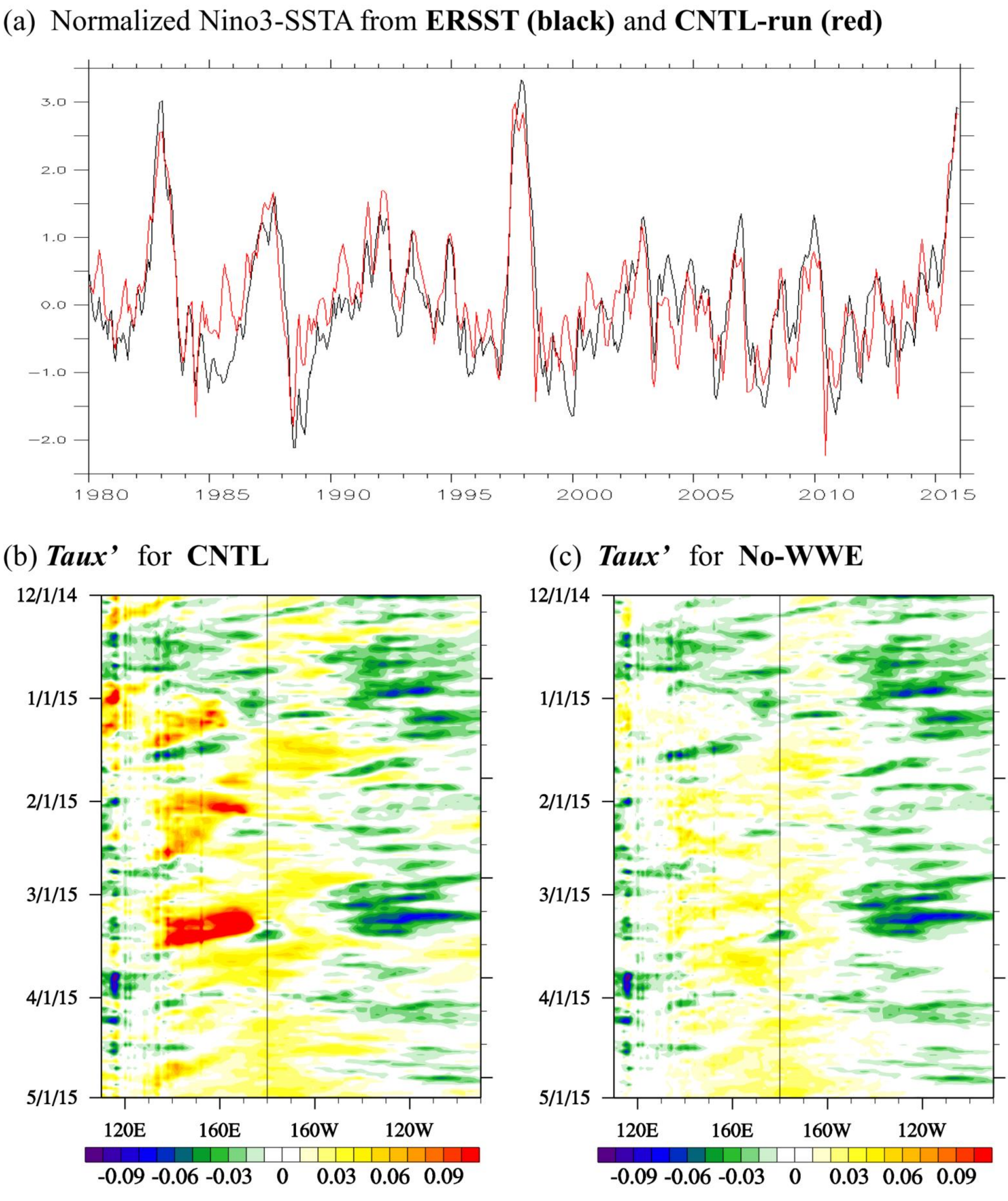

Figure S6. (a) The normalized Niño3 index from ERSST (black curve) and the CNTL run (red curve). (**b-c**) Evolution of the anomaly fields of zonal wind stress (*Taux'*) for (**b**) CNTL run and (**c**) No-WWE run. For convenient comparison, here Fig. S4b is similar as Fig. 3a. All plots were generated by the NCAR Command Language (NCL, version 6.2.1, [Software]. (2014). Boulder, Colorado: UCAR/NCAR/CISL/VETS. http://dx.doi.org/10.5065/D6WD3XH5).

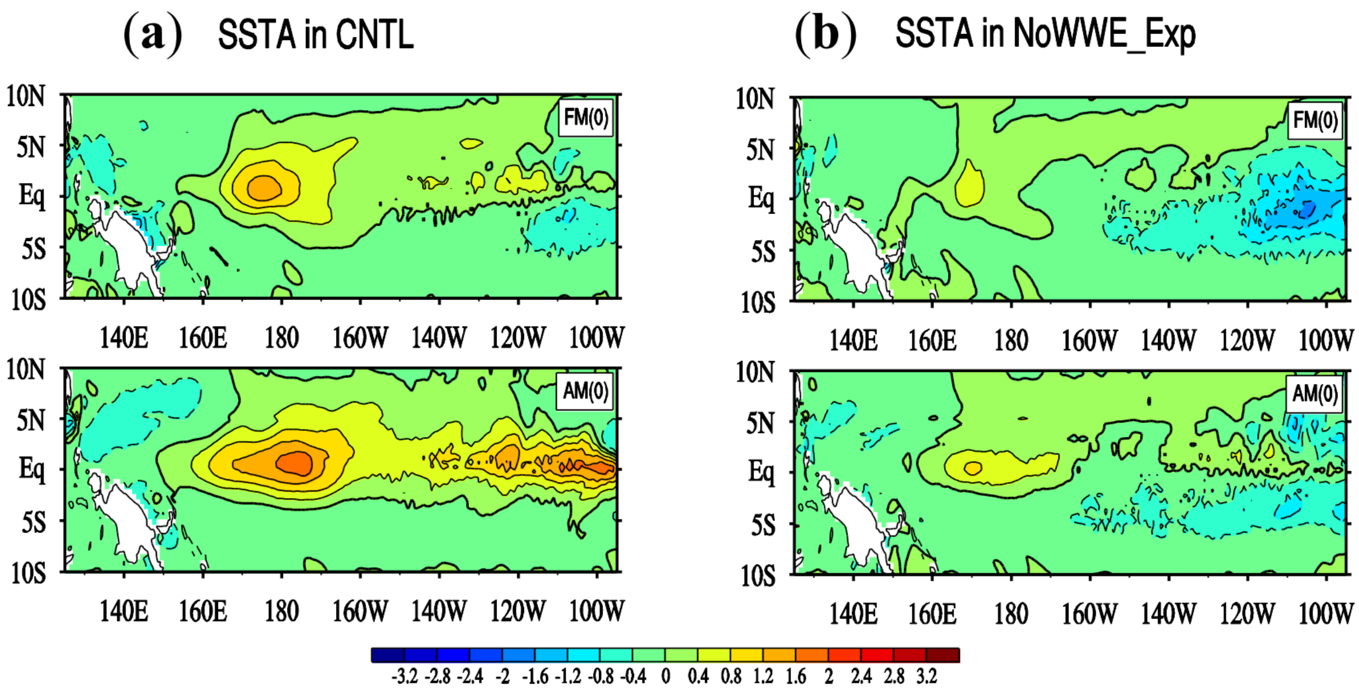

Figure S7. Time evolution of SSTA (unit: K) for February-March 2015 (FM[0]; upper) and April-May 2015 (AM[0]; bottom) in (**a**) CNTL run and (**b**) No-WWE run. All plots were generated by the NCAR Command Language (NCL, version 6.2.1, [Software]. (2014). Boulder, Colorado: UCAR/NCAR/CISL/VETS. http://dx.doi.org/10.5065/D6WD3XH5).

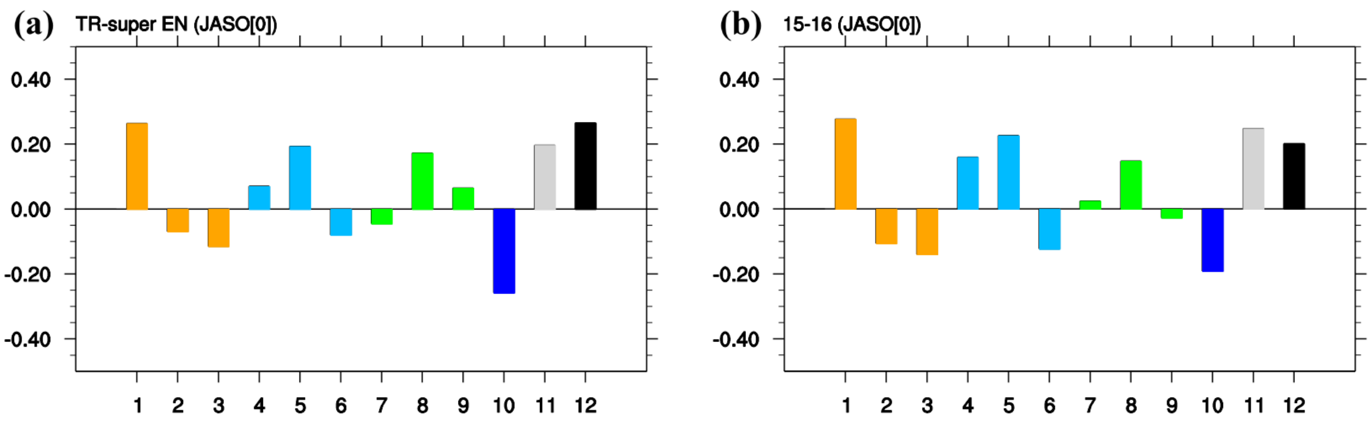

Figure S8. The mixed-layer heat budget terms (unit: K/month; averaged over JASO[0]) for Niño3 SSTA in the late developing stage of (**a**) TR-super EN and (**b**) 2015EN. All plots were generated by the NCAR Command Language (NCL, version 6.2.1, [Software]. (2014). Boulder, Colorado: UCAR/NCAR/CISL/VETS. http://dx.doi.org/10.5065/D6WD3XH5).

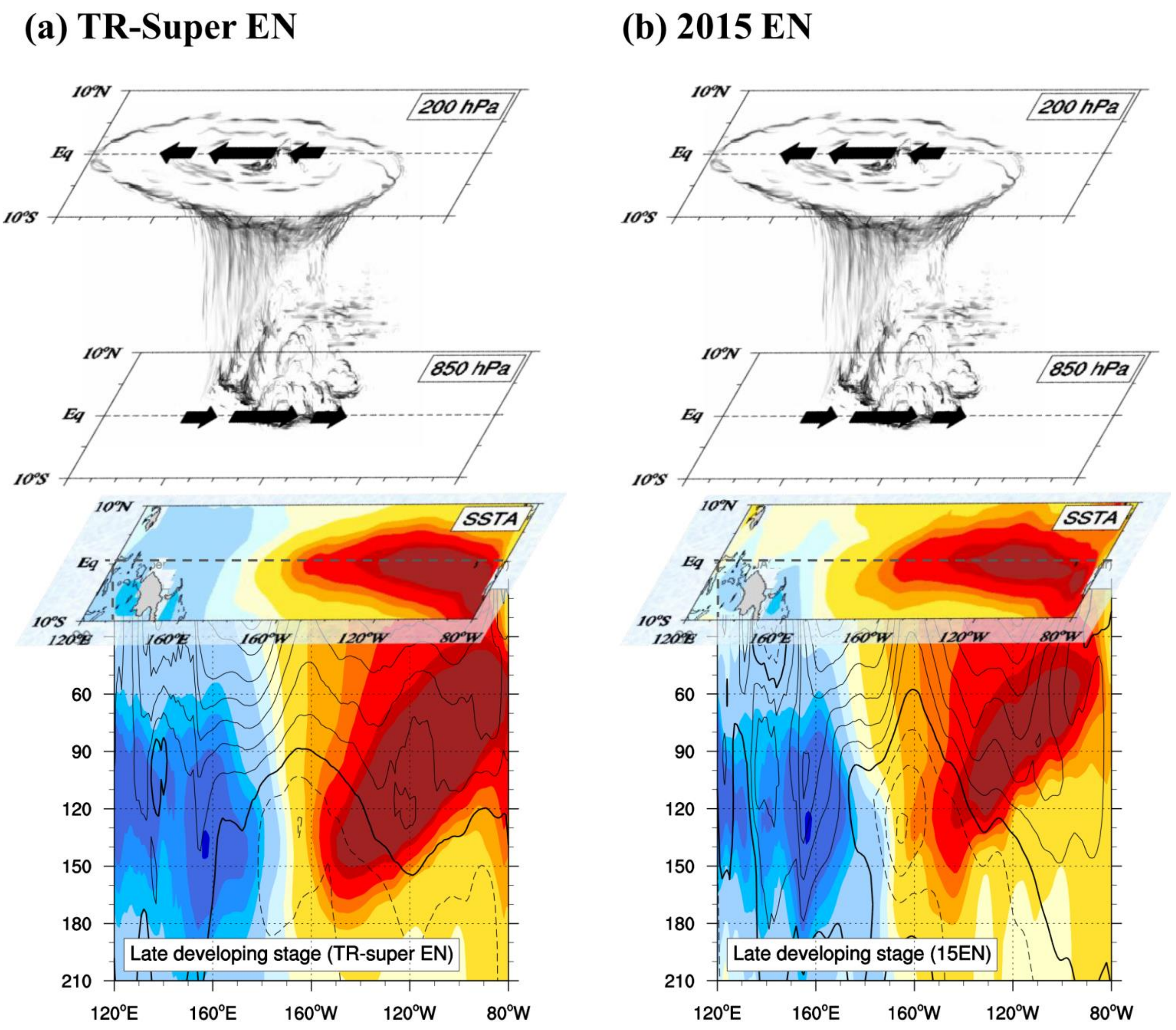

Figure S9. Schematic diagram showing that the (a) TR-super EN and (b) 2015EN exhibit similar features of the major positive air-sea feedback processes during the late developing stage (ASON[0]). The bottom panels show the equatorial profile (averaged for 5 °S-5 °N) of oceanic temperature anomaly (shading) and zonal current anomaly (contour), covered by the horizontal map of SSTA. The upper panels show anomalous westerly wind at 850 hPa with a convergence center over CEP and anomalous easterly wind at 200 hPa with a divergence center over CEP, corresponding to anomalous deep atmospheric convection. The magnitude of each variable is shown in Fig. S10. All plots were generated by the NCAR Command Language (NCL, version 6.2.1, [Software]. (2014). Boulder, Colorado: UCAR/NCAR/CISL/VETS. http://dx.doi.org/10.5065/D6WD3XH5) and the licensed Microsoft PowerPoint.

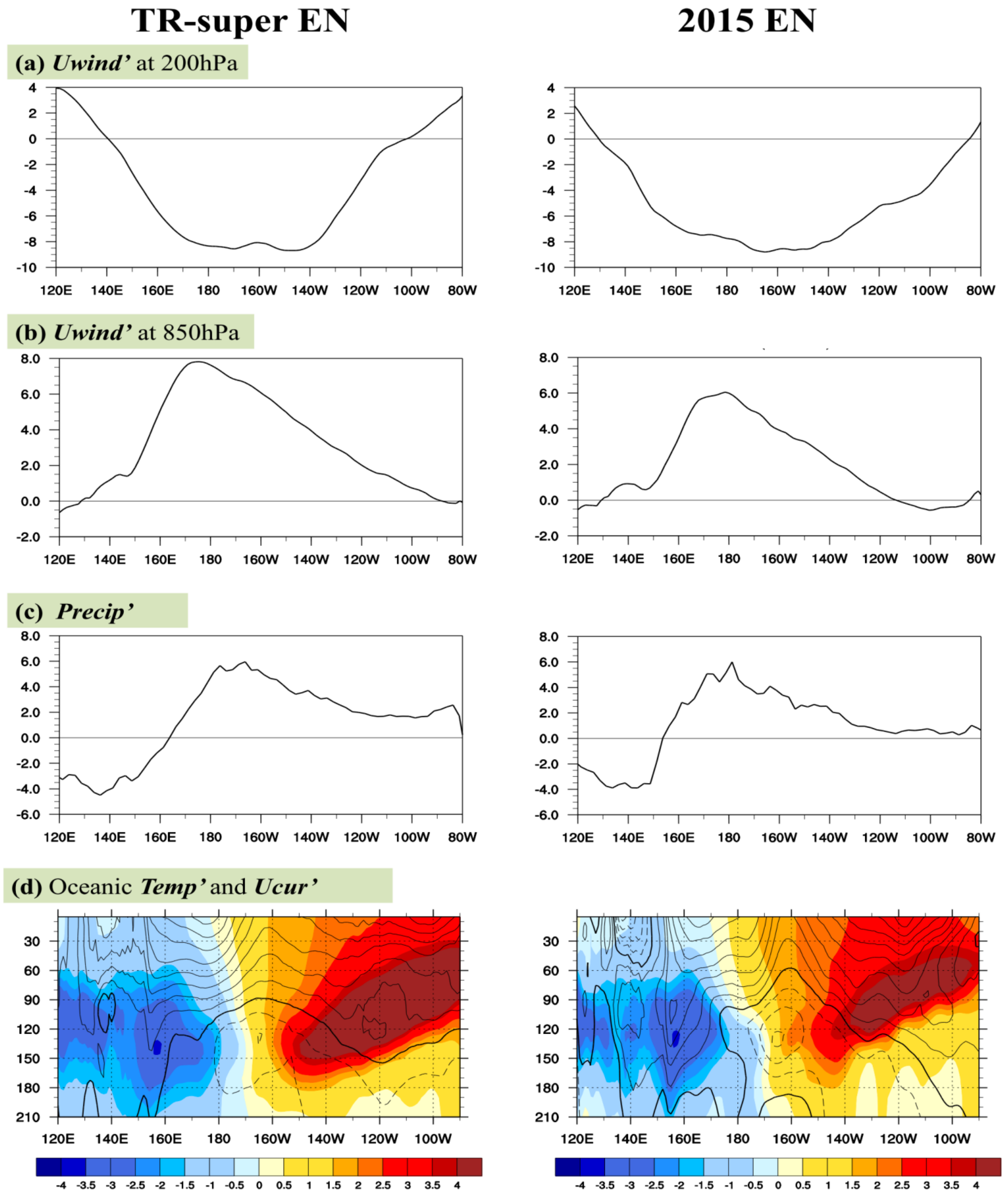

Figure S10. The main atmospheric and oceanic anomaly fields averaged over the late developing stage (ASON[0]) in the TR-super EN (left panels) and 2015EN (right panels). The upper panels show the equatorial profiles (averaged for 5°S-5°N) of anomalous zonal winds (unit: m/s) at (**a**) 200 hPa and (**b**) 850 hPa, as well as (**c**) the anomalous precipitation (unit: mm/day). (**d**) The bottom panels show the equatorial profile (averaged for 5°S-5°N) of oceanic temperature anomaly (shading; unit: K) and zonal current anomaly (contour; the interval is 0.05 m/s; solid (dashed) line indicates positive (negative) value and the solid line in bold is the zero contour). All plots were generated by the NCAR Command Language (NCL, version 6.2.1, [Software]. (2014). Boulder, Colorado: UCAR/NCAR/CISL/VETS. http://dx.doi.org/10.5065/D6WD3XH5).

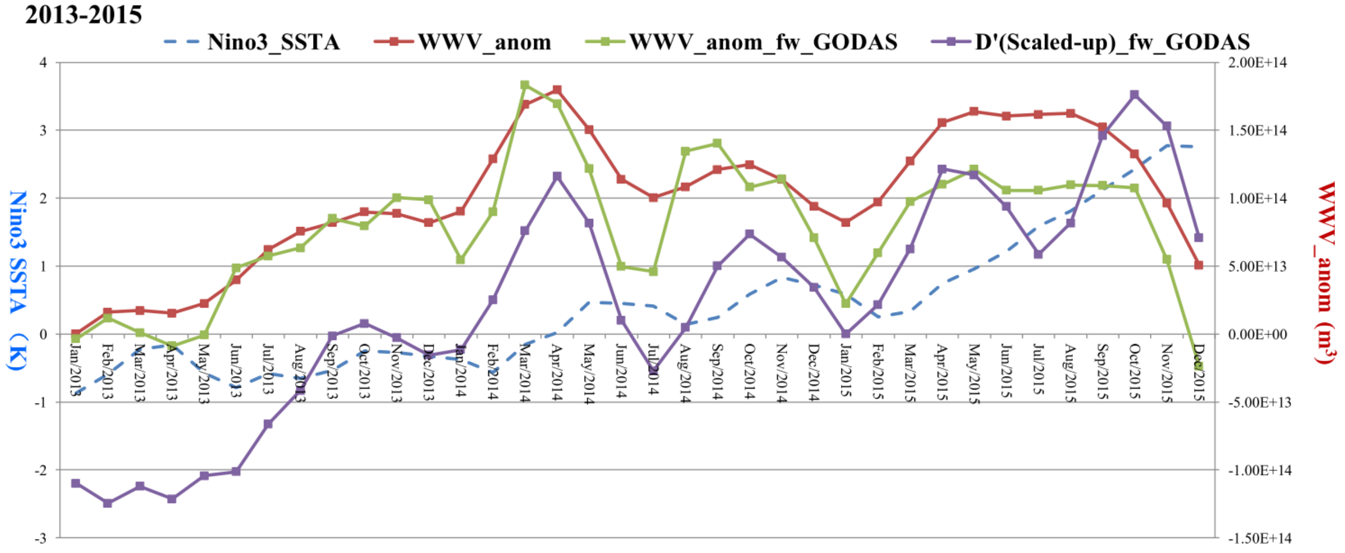

Figure S11. Evolution of Niño3 SSTA, anomalous warm water volume (WWV), and scaled-up *D'* from 2013 to 2015. The blue dashed line indicates the temporal evolution of Niño3 SSTA (left axis; unit: K). The red solid line shows the evolution of anomalous WWV (right axis; unit: m^3) from the TAO/PMEL (http://www.pmel.noaa.gov/elnino/upper-ocean-heat-content-and-enso), the green solid line shows the evolution of anomalous WWV calculated from GODAS, and the purple line shows the evolution of scaled-up *D'* from GODAS. Based on the definition in Meinen and McPhaden (2000), the integrated warm water volume (WWV) above the 20 ℃ isotherm over 5°N-5°S, 120°E to 80°W, is referred to as WWV. The scaled-up *D'* (unit: m^3) is obtained through integrating the *D'* over 5°N-5°S, 120°E to 80°W. The value of *D'* (unit: m) is estimated by using *SSH'* multiplied by $\Delta\rho / \rho_0$. This figure was generated by the licensed Microsoft Excel.
